# Occurrence of *Leishmania* infection in the immediate geographic region of Ji-Paraná, Rondônia State, Brazil

**DOI:** 10.1590/0037-8682-0212-2021

**Published:** 2021-08-20

**Authors:** Aliny Pontes Almeida, Paula Frassinetti Medeiros de Paulo, Antonio Marques Pereira, Cinthya de Andrade Gujanwski, Valéria Ferreira, Glaucilene da Silva Costa, Moreno Magalhães de Souza Rodrigues, Ricardo de Godoi Mattos Ferreira, Jansen Fernandes Medeiros

**Affiliations:** 1 Centro Universitário São Lucas, Curso de Medicina Veterinária, Ji-Paraná, RO, Brasil.; 2 Fundação Oswaldo Cruz, Fiocruz Rondônia, Porto Velho, RO, Brasil.; 3 Instituto Nacional de Epidemiologia da Amazônia Ocidental (INCT-EpiAMO), Porto Velho, RO, Brasil.; 4 Fundação Universidade Federal de Rondônia, Programa de Pós-Graduação em Biologia Experimental, Porto Velho, RO, Brasil.

**Keywords:** American Cutaneous Leishmaniasis, Rondônia, Epidemiology

## Abstract

**INTRODUCTION:**

This study evaluated the epidemiology of American cutaneous leishmaniasis in the immediate region of Ji-Paraná, Rondônia State.

**METHODS:**

Samples and epidemiological data were collected from 105 patients.

**RESULTS:**

*Leishmania* infection was observed in 58 (55.2%) patients, and *Leishmania braziliensis* was present in 82.9% of the 41 sequenced samples. Infected patients were predominantly male (93.1%). *Leishmania* infection was twice as prevalent among rural inhabitants versus urban inhabitants. Lesions were more frequent in the upper limbs (arms/hands, 41.82%).

**CONCLUSIONS:**

The present data corroborate the zoonotic profile of cutaneous leishmaniasis; this information could help to improve surveillance and control strategies.

Leishmaniasis, a disease caused by *Leishmania*, is widely distributed worldwide. Brazil is one of the six countries with the highest rates of both American cutaneous leishmaniasis (ACL) and visceral leishmaniasis (VL), with approximately 20,000 and 3,400 reported cases per year, respectively^1^.

The North Region of Brazil has experienced a particularly high number of leishmaniasis cases; a total of 6,681 ACL cases and 496 VL cases were reported in 2019 alone^1^. ACL is highly endemic in Rondônia State, and the primary source of human infection is the bite from infected female sand flies in forest environments. Rondônia records an average of 1,000 cases per year, with an incidence rate of 45 cases per 100,000 inhabitants. Seven *Leishmania* species have been recorded as agents of infection, with *Leishmania braziliensis* (Vianna) being the most prevalent^2^.

Rondônia is geographically divided into six immediate regions: Porto Velho, Ariquemes, Jarú, Ji-Paraná, Cacoal, and Vilhena (Figure 1B). In the immediate region of Ji-Paraná, where this work was conducted, 139 cases of ACL were registered in 2019 alone^1^. Despite the high incidence of ACL in Rondônia, studies on leishmaniasis in patients have been limited to the municipalities of Porto Velho and Monte Negro^2^ ^,^ ^3^. Thus, the present study aimed to identify the *Leishmania* species responsible for ACL cases in the immediate region of Ji-Paraná and to use the data collected from diagnosed patients to determine the epidemiological profile of ACL in this region.

The immediate region of Ji-Paraná is composed of 13 municipalities located in the central region of Rondônia (Figure 1C). Human samples (skin lesions) were collected between December 2016 and November 2018 in Ji-Paraná, at Padre Adolfo Lutz Tropical Disease Research Institute. This institute is the reference center responsible for assisting and diagnosing suspected ACL cases. The institute performs direct examination using a sterile scalpel to collect samples from the edge of ulcerated lesions (scarification). The collected sample material was smeared on slides, stained with Giemsa, and observed at 100X magnification using optical microscopy.

**FIGURE 1:**
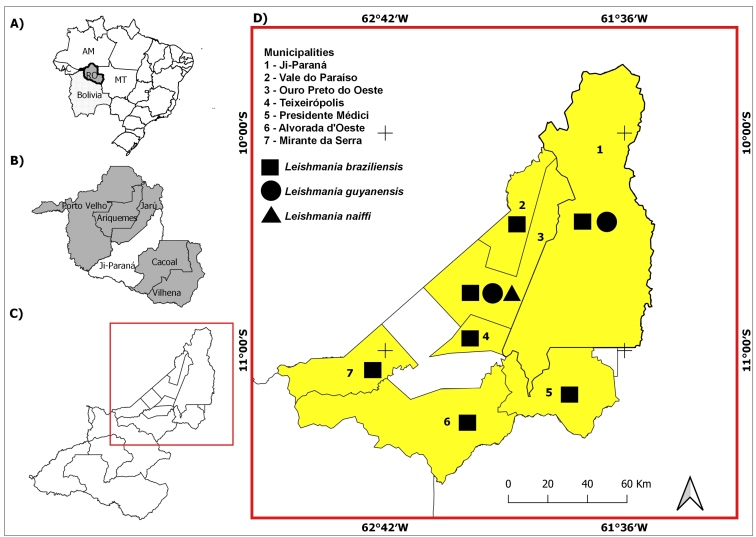
**(A)** Geographic location of Rondônia State; **(B)** Immediate regional divisions of Rondônia State according to the IBGE (2017); **(C)** Municipalities that comprise the immediate region of Ji-Paraná; **(D)** Municipalities and *Leishmania* species detected.

Patients with skin lesions were invited to participate in the study, and those who agreed to receive and signed the Free and Informed Consent Form (ICF). Subsequently, a trained technician from the institute collected biological samples from the participating patients. Samples were stored in 50 µL of phosphate-buffered saline (PBS), identified, and frozen at -20 °C for subsequent DNA extraction. Patients also answered an epidemiological questionnaire that identified their sex; age; living area (rural or urban); their involvement in hunting, fishing, or eco-tourism; and the proximity of their home to forests. Moreover, a clinical examination was performed on all patients to collect data on the presence, number, and location of lesions.

DNA extraction was performed using the Illustra Blood Mini Spin Kit (GE Healthcare Life Sciences®, Buckinghamshire, United Kingdom). Polymerase chain reaction (PCR) assays targeted kDNA, according to the protocol of Oliveira et al. (2005)^4^, using the primers 5′-GGG(G/T)AGGGGCGTTCT(G/C)CGAA-3′ and 5′-(G/C)(G/C)(G/C)(A/T)CTAT(A/T)TTACACCAACCCC-3′ (120 pb). Positive samples were subjected to PCR targeting the hsp70 region using the primers 5′-GGACGAGATCGAGCGCATGGT-3′ and 5′-TCCTTCGACGCCTCCTGGTTG-3′ (240 bp)^5^. The PCR products were checked on a 2% agarose gel and purified using the ExoSAP-IT™ PCR Product Cleanup Reagent (Thermo Fisher Scientific, California, USA). The purified PCR products were sequenced at the Fiocruz sequencing facility (RTP01E).

The obtained sequences were visualized and edited using Phred, Phrap, and Consed software and then submitted to BLASTn (http://blast.ncbi.nlm.nih.gov/Blast.cgi) and compared with sequences from GenBank (http://www.ncbi.nlm.nih.gov/genbank/).

Epidemiological data were analyzed using descriptive statistics. For categorical variables, we computed the data as proportion and percentage of patients in each group. For numerical variables, we computed the data as median and interquartile range (IQR). The odds ratio (OR) of infection for *Leishmania* was obtained for its association with sex; age; living area (rural or urban); their involvement in hunting, fishing, or eco-tourism; and the proximity of their home to forests. We computed the OR using conditional maximum likelihood estimation. All analyses were performed using the free program language Python 3.8. Data and codes have been provided as supplementary files **(** Supplementary TABLE **).**

From December 2016 to November 2018, a total of 105 lesion samples were collected from patients who were suspected to have cutaneous leishmaniasis infection and were residents of Ji Paraná, Presidente Médici, Ouro Preto do Oeste, Vale do Paraíso, Alvorada do Oeste, or Mirante da Serra **(** Figure 1D**)**. Most patients were male (85.71% [90/105]), and their average age was 39 years (IQR: 30-49 years) for men and 34 years (IQR: 29-48 years) for women. The male/female ratio was 6.00 (85.7% male, 90/105; 14.3% female, 15/105).

Of the 105 patients examined, 58 tested positive (55.2%) for *Leishmania* spp. using parasitological and PCR methods. The lowest number of cases occurred in individuals above 50 years of age (13.80%; 8/58); the prevalence began to increase in the 20-30 year age group (24.14%; 14/58), and the prevalence was highest in the 31-40 year group (27.60%; 16/58) and in the 41-50 year age group (27.60%; 16/58). Of the 58 positive cases, 41 samples were sequenced, and the following species were identified: *Leishmania braziliensis* (82.90%; 34/41), *Leishmania guyanensis* (Floch) (12.20%, 5/41), and *Leishmania naiffi* (Lainson & Shaw) (4.90%*;* 2/41). *Leishmania braziliensis* was the most prevalent species and was found in patients from every municipality. *Leishmania guyanensis* was detected in patients from the municipalities of Ji-Paraná and Ouro Preto do Oeste, and *L*. *naiffi* was detected solely in patients from the municipality of Ouro Preto do Oeste **(** Figure 1D**)**.

According to our data, *Leishmania* prevalence was higher in men: 93.10% (54/58) of the positive samples belonged to men; thus, the OR of being positive for *Leishmania* was highest in this group. Individuals from rural areas were more likely (OR: 2.27) to be positive for *Leishmania* than those who lived in urban areas (Table 1). Furthermore, our data indicate that individuals whose primary occupation is ecotourism were approximately twice as likely to be positive for *Leishmania* relative to individuals who were in other occupations (Table 1).

**TABLE 1:** Cutaneous leishmaniasis cases by sex, area, activity, and odds and confidence interval (CI) in the immediate region of Ji-Paraná, Rondônia State, during 2016-2018.

Variables	Negative %	Positive %	OR (95% CI)
**Sex**			
Male	76.50	93.10	4.07
Female	23.40	6.10	Reference
**Living area** ^a^			
Urban	70.20	50.10	Reference
Rural	25.55	40.40	2.27
Mixed date	4.25	8.80	2.80
**Hunting activity** ^b^			
Yes	26.70	28.90	1.10
No	73.30	71.20	
**Lives near the forest**			
Yes	54.50	64.15	1.48
No	45.50	35.85	
**Practice eco tourism** ^c^			
Yes	19.00	41.50	2.99
No	81.00	58.50	
**Practice fishing** ^d^			
Yes	61.70	76.35	2.00
No	38.30	23.65	

^a^ Living area information was missing for 1 individual

^b^ Hunting activity information was missing for 8 individuals

^c^ Practice ecotourism information was missing for 5 individuals

^d^ Fishing information was missing for 3 individuals.

OR: odds ratio; CI: confidence interval

All positive patients presented with the clinical cutaneous form of leishmaniasis, and the majority presented only one lesion (94.83%; 55/58). Of the 55 patients with only one lesion, the body sites most affected were the arms/hands (41.82%; 23/55) and legs/feet (38.18%; 21/55) (Figure 2).

**FIGURE 2:**
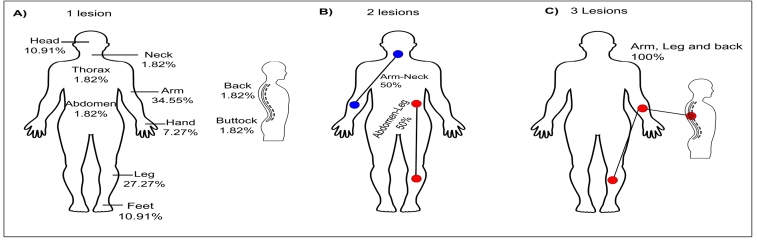
Clinical features of cutaneous leishmaniasis in relation to the number of lesions and body sites affected.

This study delineates the epidemiological framework of CL in the immediate region of Ji-Paraná. The study highlighted a pattern of *L*. *braziliensis* infection in seven municipalities in this region. Indeed, this is the most prevalent species in Brazilian cases; human cases have been reported in every state of the Brazilian Amazon, and the prevalence of this *Leishmania* species is highest in states such as Acre, Pará, and Roraima^6^ ^,^ ^7^. In Rondônia, *L. braziliensis* has already been registered in the immediate regions of Porto Velho and Ariquemes^2^ ^,^ ^3^, as well as in the Ji-Paraná region. *Leishmania braziliensis* is a common species widely distributed in Brazil, and this success could be related to a variety of sand fly species that have vector competence for this protozoan^8^. In Rondônia, there is an abundance of *Psychodopygus davisi* (Root) and *Psychodopygus hirsutus hirsutus* (Mangabeira), which have been detected with promastigote forms and *Leishmania* DNA^9^ ^,^ ^10^, and may contribute to the transmission cycle of the parasite in the region.

We observed two cases of *L*. *guyanensis* in two municipalities; its prevalence in Rondônia is low relative to that in *L. braziliensis* ^2^ ^,^ ^3^. *Leishmania guyanensis* is widely distributed in the Amazon Basin, and its distribution is certainly related to the presence and abundance of *Nyssomyia umbratilis* (Ward & Fraiha) populations^11^ ^,^ ^12^ ^,^ ^13^. In Amazonas, *L. guyanensis* is the primary agent of ACL, and *N*. *umbratilis*, its main vector, is present in high abundance^13^. Studies indicate that *N. umbratilis* is present in low abundance in Rondônia^9^ ^,^ ^10^.

The presence of *L*. *naiffi* DNA in two samples from Ouro Preto do Oeste demonstrates that this species is in circulation in the region and that human hosts play a role in its transmission cycle. Information about the presence of this species in Rondônia is sparse; this species has been reported in sand fly species^4^ and in only one human. This may be due to the fact that *L*. *naiffi* occurs at a low frequency and has low pathogenicity in Rondônia^14^. Consequently, people may not seek health assistance, which would cause the distribution of *Leishmania* species such as *L*. *naiffi*, *L*. *guyanensis*, and *L*. *braziliensis* to be underestimated, which could have affected the results.

In the immediate region of Ji-Paraná, data indicated that the probability of *Leishmania* infection was double among rural inhabitants versus urban inhabitants, that infections were most prevalent among people aged 30-50 years old, and that infections were more common in men than in women. This profile could be explained by the fact that people aged 30-50 years are the most economically active, and that rural residents generally live and work near forests or forest fragments, which greatly increases their exposure to sand fly bites. This pattern has been observed in Rondônia and other states in the Brazilian Amazon^9^ ^,^ ^10^ ^,^ ^11^ ^,^ ^12^ ^,^ ^13^ ^,^ ^14^.

The highest proportion of infections occurred as single lesions, and the parts of the body most affected were the arms, legs, head, and feet, which corroborate the findings of other studies^12^ ^,^ ^13^ ^,^ ^14^. These parts of the body are the most affected because they remain exposed to vectors for many hours of the day when individuals work in forest environments (primarily in the occupations of logging and hunting)^7^ ^,^ ^11^ ^,^ ^12^.

A pattern of infection was also observed in people who practiced ecotourism but did not live in rural areas. In Rondônia, the presence of cases in urban areas could be attributable to cultural practices such as farm visits, camping, and fishing during holidays. People who practice these leisure activities generally remain in close proximity to forest environments for many hours; therefore, their risk may have increased exposure to sand fly bites and possible infection by *Leishmania* species^13^.

In summary, the obtained data indicate a zoonotic profile of the ACL in the immediate region of Ji-Paraná, in which people present lesions primarily on the exposed parts of the body. Our data also demonstrate that the likelihood of ACL infection may be related to sex, place of residence, and occupation because, for example, living in a rural area increases the OR of being positive for *Leishmania.* The prevalence of *Leishmania* species may be related to sand fly vectors and reservoir distributions, but studies conducted in the future will need to evaluate these relationships. All information presented herein could help to improve surveillance and control strategies aimed at reducing the number of CL cases in the municipalities of the immediate region of Ji-Paraná and in other regions of Rondônia State.

## Supplementary Material

**TABLE:** *Leishmania* species identified of sequences obtained from skin lesions of humans in Ji-Paraná Immediate Region.

Species	Identity (%)	Query cover (%)	E-value	Genbank Acession
*Leishmania braziliensis*	99.1	100	0	HF586369
*Leishmania naiffi*	99,5	100	0	GU071183
*Leishmania guyanensis*	99,8	100	0	HF586362
*Leishmania guyanensis*	100	100	0	FN395051
*Leishmania guyanensis*	99,9	99,92	0	FN395051
*Leishmania braziliensis*	99.8	100	0	LS997627
*Leishmania braziliensis*	100	100	0	LS997627
*Leishmania braziliensis*	100	100	0	FN395039
*Leishmania braziliensis*	99.8	100	0	FR715986
*Leishmania braziliensis*	99.8	100	0	FR715986
*Leishmania braziliensis*	99.8	100	0	FR715986
*Leishmania braziliensis*	99.8	100	0	FR715986
*Leishmania braziliensis*	99.9	100	0	HF586369
*Leishmania braziliensis*	100	100	0	HF586369
*Leishmania braziliensis*	99.9	100	0	HF586369
*Leishmania braziliensis*	99.9	100	0	HF586369
*Leishmania braziliensis*	99.9	100	0	HF586369
*Leishmania braziliensis*	99.9	100	0	HF586369
*Leishmania braziliensis*	99.9	100	0	HF586369
*Leishmania braziliensis*	99.9	100	0	HF586369
*Leishmania braziliensis*	99.9	100	0	HF586369
*Leishmania braziliensis*	99.9	100	0	HF586369
*Leishmania braziliensis*	99.9	100	0	HF586369
*Leishmania guyanensis*	98.7	100	1.30E-156	MN688569
*Leishmania guyanensis*	100	100	0	FN395051
*Leishmania naiffi*	99.5	100	0	GU071183
*Leishmania braziliensis*	99.2	100	1.07E-126	HF586369
*Leishmania braziliensis*	100	100	1.03E-167	MF687097
*Leishmania braziliensis*	100	99.25	0	MF344845
*Leishmania braziliensis*	98	100	3.94E-95	MW094222
*Leishmania braziliensis*	99.1	100	3.31E-106	XM_001566273
*Leishmania braziliensis*	100	100	3.22E-106	MN395479
*Leishmania braziliensis*	100	100	8.62E-102	MN395479
*Leishmania braziliensis*	99.5	100	5.10E-99	MW094222
*Leishmania braziliensis*	99	100	6.59E-98	MW094222
*Leishmania braziliensis*	99	100	3.01E-96	MW094222
*Leishmania braziliensis*	100	100	1.97E-108	MN395479
*Leishmania braziliensis*	99.5	100	5.10E-99	MW094222
*Leishmania braziliensis*	100	100	1.97E-108	KY249628
*Leishmania braziliensis*	100	100	1.15E-105	MW094222
*Leishmania braziliensis*	99	100	4.06E-100	MW094222
